# Experiences of health care providers with integrated HIV and reproductive health services in Kenya: a qualitative study

**DOI:** 10.1186/1472-6963-13-18

**Published:** 2013-01-11

**Authors:** Richard Mutemwa, Susannah Mayhew, Manuela Colombini, Joanna Busza, Jackline Kivunaga, Charity Ndwiga

**Affiliations:** 1 Department of Population Health, London School of Hygiene & Tropical Medicine, Keppel Street, London, WC1E 7HT, UK; 2 Population Council, Nairobi, Kenya; 3 Department of Global Health and Development, London School of Hygiene & Tropical Medicine, London, UK

**Keywords:** Integration, HIV/AIDS, Reproductive health, Health care providers, Challenges, Benefits

## Abstract

**Background:**

There is broad consensus on the value of integration of HIV services and reproductive health services in regions of the world with generalised HIV/AIDS epidemics and high reproductive morbidity. Integration is thought to increase access to and uptake of health services; and improves their efficiency and cost-effectiveness through better use of available resources. However, there is still very limited empirical literature on health service providers and how they experience and operationalize integration. This qualitative study was conducted among frontline health workers to explore provider experiences with integration in order to ascertain their significance to the performance of integrated health facilities.

**Methods:**

Semi-structured in-depth interviews were conducted with 32 frontline clinical officers, registered nurses, and enrolled nurses in Kitui district (Eastern province) and Thika and Nyeri districts (Central province) in Kenya. The study was conducted in health facilities providing integrated HIV and reproductive health services (post-natal care and family planning). All interviews were conducted in English, transcribed and analysed using Nvivo 8 qualitative data analysis software.

**Results:**

Providers reported delivering services in provider-level and unit-level integration, as well as a combination of both. Provider experiences of actual integration were mixed. At personal level, providers valued skills enhancement, more variety and challenge in their work, better job satisfaction through increased client-satisfaction. However, they also felt that their salaries were poor, they faced increased occupational stress from: increased workload, treating very sick/poor clients, and less quality time with clients. At operational level, providers reported increased service uptake, increased willingness among clients to take an HIV test, and reduced loss of clients. But the majority also reported infrastructural and logistic deficiencies (insufficient physical room space, equipment, drugs and other medical supplies), as well as increased workload, waiting times, contact session times and low staffing levels.

**Conclusions:**

The success of integration primarily depends on the performance of service providers which, in turn, depends on a whole range of facilitative organisational factors. The central Ministry of Health should create a coherent policy environment, spearhead strategic planning and ensure availability of resources for implementation at lower levels of the health system. Health facility staffing norms, technical support, cost-sharing policies, clinical reporting procedures, salary and incentive schemes, clinical supply chains, and resourcing of health facility physical space upgrades, all need attention. Yet, despite these system challenges, this study has shown that integration can have a positive motivating effect on staff and can lead to better sharing of workload - these are important opportunities that deserve to be built on.

## Background

There is a broad consensus on the value of integration of HIV services and reproductive health services, particularly in regions of the world with generalised HIV/AIDS epidemics and high reproductive morbidity [1-4]. These communities tend to have less effective health services that do not adequately meet local needs. To address that service deficit, integration of reproductive health services has been a central goal of most health systems since the 1994 Cairo International Conference on Population and Development (ICPD) [5]. Integration is thought to increase access to and uptake of health services; and improves their efficiency and cost-effectiveness through better use of available resources [6-10]. In the case of HIV and reproductive health services, the basic argument is that integration has the potential to improve uptake of either reproductive health services, HIV services, or both.

Yet, the shift from provision of vertical to integrated care is a complex transformation that demands significant adjustments in almost all major aspects of health care organisation. As Zotti *et al.*[11] observe, employee behaviour is central to organisational change and ultimately determines the outcome of any change process. Generally, there are two possible levels at which providers behavioural responses to integration may be influenced. First, at individual level, where direct benefits to the individual and their personal aspirations are priority, the way providers perceive integration (as a threat to job security or as an opportunity to advance their professional skills) may influence their delivery of services [12]. Second, at the operational level where, in delivering an integrated service, providers may experience systemic improvements or challenges that accompany an integrated health service [1,8,10,13-15]. However, there is still very limited empirical literature on health service providers and how they experience and operationalize integration. This evidence is key to the successful introduction and sustainability of integration into a health system.

This paper is based on a qualitative component of a large intervention research project, *Integra*, implemented in Kenya and Swaziland to determine the effectiveness of integrating HIV/AIDS into family planning (FP) and post-natal care (PNC) services in primary level health facilities. The main intervention study was quasi-experimental, with intervention facilities implementing integrated services and comparison facilities providing standard care. All providers were trained on the Balanced Counselling Strategy Plus (BCS+) an algorithm that takes the provider through 4 standardised stages of care provision in a session with a client [16]. The training strategy used was mentorship, where experienced senior staff were trained and then over time allowed to mentor their junior staff. Study coordinators regularly conducted support and assessment visits during which individual providers were tested to determine their attained skill level. Actual implementation of the study only begun after providers in the study facilities had been certified to have attained a pre-determined minimum level of clinical skill. Continued support was provided by responsible coordinators in the study. At the time of conducting the study, facilities in both Eastern and Central provinces had been implementing integrated reproductive health services for at least 5 months. By agreement with the Governments of Kenya and Swaziland, initial clinical supplies were provided to study facilities, after which routine government medical supply systems took over. Throughout the study there was regular contact with the local Ministries of Health in both countries.

This qualitative study was conducted among frontline health care workers to explore both individual and operational level provider experiences with integration. The purpose of this qualitative study was not to evaluate the impact of the intervention on providers but to better understand the various operational contexts of integration during the research, as well as establish the extent to which any challenges mediated the beneficial attributes of integrated services.

## Methods

The qualitative study was conducted in Kitui district in Eastern Province and Thika and Nyeri districts in Central province in Kenya. Demographic profiles across districts are comparable. There was uniformity in the package of integrated services implemented in each province, although there was a different service focus in each province. In Eastern province (Kitui), the study was conducted in health facilities implementing integrated HIV and PNC services. In Central province (Thika and Nyeri), the study was conducted in health facilities implementing integrated HIV and FP services.

Ethical approval was granted for the study locally in Kenya by the Kenya Medical Research Institute (Reference: NON/SSC/113); as well as by the London School of Hygiene & Tropical Medicine Ethics Committee (Reference: 5426). Each respondent provided written informed consent to participate in the study and be interviewed.

The qualitative study used semi-structured indepth interviews (IDIs) to collect experiential data from frontline health care providers in the selected hospitals, sub-district hospitals and health centres in each study district. In total 32 IDIs were conducted with health care providers between late June and early July 2010. Table 1 presents basic characteristics of the interviewees. In order to provide some technical context to the findings, respondents were asked to describe what services they provided in their individual role and *how* they provided those services, in order to determine the range of integration models operating in the facilities.

**Table 1:** Basic characteristics of interviewed health care providers

**Characteristic**	**Number**
**Province:**	
Central	18
Eastern	14
**Sex:**	
Males	6
Females	26
**Profession:**	
Clinical Officers	3
Registered Nurses	16
Enrolled Nurses	13
**Facility Level:**	
District Hospital	10
Sub-District Level	11
Health Centre	11

Median total number of years served at facility of interview by the provider: 2.5 years (range: 0.5 years – 28 years).

All IDIs were conducted in English and transcribed verbatim. Transcriptions were performed by a small team of research assistants selected from the broader group that conducted the field interviews. The electronic transcripts were then loaded into Nvivo 8 qualitative data software [17], for analysis. Analysis was based on a combination of both thematic and inductive free-coding.

## Results

### Integration models in operation

Our analysis showed that three forms of operational integration were reported by providers who participated in the study (Table 2).

**Table 2:** **Three different operational models of integration in*Integra*study facilities in Kenya based on providers’ reports**

**Operational integration model**	**Provider(s) client can receive service(s) from in one visit**	**Room(s) client can receive service(s) from in one visit**
*Provider-level integration*	Client receives all required services from one provider	Client receives required services in one room
*Unit-level integration*	Client receives required services from different specialist providers	Client receives required services in different rooms
	*Client may receive more than one service within each specialist unit*	
*‘Mixed’ integration*	Client receives required services from one provider	Client receives required services in different rooms

In *provider-level integrated* facilities, providers reported providing more than one service to a client in the same room. In facilities with good numbers of staff, this meant a facility having more than one provider delivering an almost identical package of services in separate rooms. In poorly staffed facilities it was one or two staff each providing a range of services.

Before integration we used to offer our services in different places but nowadays we offer them all together in the same room, through this we are able to provide our services [better]. (Community Health Nurse, Health Centre, Thika)

In *unit-level integrated* facilities, providers reported that clients receive integrated services within specialist units under different roofs within the same facility. For instance, in Kenya government policy has more recently driven a steady increase in HIV/AIDS-focused comprehensive care clinics (CCCs) being built at primary level health facilities, as a way to minimise numbers of HIV/AIDS clients being referred between facilities.

Instead of referring clients to another building or health centre, you make sure all the services are close (together) in one place. Sometimes when we are short of staff I have to move from one room to the next to see clients waiting there, but at least it’s just nearby… even for the client to walk. (Clinical Officer, Health Centre, Nyeri)

A third less common form of integration reported seemed to be some kind of *blend* of the two above. A few providers reported providing more than one service per client but in different rooms. In this integration model, a health care provider moved with the client from one room to another in order to provide a number of services which, mainly for reasons of limited room space and equipment, could not be delivered in one room.

Here because of shortage of rooms most times I have move with my patient from one examination room to the next where there is equipment. We need more rooms. (Enrolled Nurse, Sub-District Hospital, Kitui)

In discussion of their experiences of delivering integrated services, in whatever format, our respondents identified a range of benefits and challenges; these are now discussed.

### Benefits of integration

Table 3 summarises the benefits of integration reported by providers. The majority of providers said that integration has benefited them and their clients. The benefits of integration may be grouped into individual level and operational level benefits.

**Table 3:** Summary of benefits and challenges of integration reported by providers

**Benefits**	**Challenges**
***Increased job satisfaction:***	***Poor work conditions & support:***
•Increased client satisfaction	•Low salaries
•Personal skills enhanced	•Lack of psychosocial support for occupational stress management
•Experiential learning	
•Professional stimulation	
***Improved communication, performance & systems:***	***Lack of systems adaptation to support integration:***
•Improved communication among staff	•Increase in workload per provider
•Increase in client repeat visits	•Burdensome clinical recording
•Increase in service uptake	•Long session times
•No more multiple queues per visit for the client	•Long waiting times for clients
	•Lack of clinical supplies, equipment, room-space, and erratic water & electricity supply
	•Lack of guidelines on user-fee management
•Increase in willingness to take HIV test among clients	
•*Convenience*: reduced room-to-room movement by staff during service provision	
•Decrease in numbers of clients who leave before being attended during a visit	
•Reduced pressure on under-staffed facilities	
•Reduced workload per provider	

### Increased job satisfaction

Many providers reported that integration enhanced job satisfaction by providing a better quality service, which led to their receiving more regular positive feedback from clients.

I think with integration, you are able to serve the client better and capture each and every detail of a patient (holistically). (The client) will not go home with a certain problem unattended. That is very satisfying. (Enrolled Nurse, Health Centre, Kitui)

Clients come praising you, when you meet them outside (the facility). They say that your health service has really improved because you do not keep on sending us here and there causing stigma. (Enrolled Nurse, Hospital, Thika)

Professionally, integration was said to enhance provider skills through training and the broader environment of clinical experience in which staff currently operated. Providing more than one service not only broadened their skills but also increased their awareness about other health problems.

I am more competent in the way I offer my services, I have the knowledge in every method, I have the knowledge to administer methods like the IUCD and jadelle which I never knew before, I have been able to treat people under one roof without referring them. (Enrolled Nurse, Sub-District Hospital, Thika)

..because I'm able to see more clients than I used to and get more experience… because I'm dealing with different clients with different issues…it is building me as a nurse, profession-wise. So I'm more satisfied. (Registered Nurse, Sub-District Hospital, Kitui)

Constantly changing clientele, and confronting new health problems in an integrated facility, provided greater professional stimulation and reduced the dreariness that often characterises vertical ‘silo’ services.

…where there is no integration there is that boredom because of doing one thing and there is no change. In integration, you are giving this injection now, the next patient will come in with a certain service to be provided so it changes, it keeps on rotating in your mind…and you enjoy the work. It boosts my morale, because the monotony is not there. (Enrolled Nurse, Hospital, Nyeri)

#### Improved communication, performance and systems

One of the commonly mentioned positive changes due to integration was that overall attitudes towards work and working relationships had improved among staff in the facilities. There may be several explanations for that, but some providers felt that since integration communication among staff in facilities had greatly improved.

Nowadays we communicate…and that’s been really helpful I think. You don’t feel alone on the job. It never used to happen before. (Enrolled Nurse, Health Centre, Kitui)

Providers observed that due to integration service uptake and overall client numbers had increased, and that facilities were seeing more clients coming back for repeat visits due to satisfaction.

Client numbers have definitely gone up… (For example) if you are working in the (child welfare clinic) you can now (expect to) see around 100 children per day… (Enrolled Nurse, Hospital, Thika)

More clients are coming back… I think because, when you see a client in one room, and you’re able to give her the services she wanted, she’ll come back. (Registered Nurse, Sub-District Hospital, Kitui)

Providers felt that clients benefited directly from integration because they no longer had to queue three or four times at different provider rooms per visit to receive all the services they wanted; and that majority clients were now receiving more than just the one service for which they attended a facility per visit. In addition, more clients were now willing to be tested for HIV due to enhanced privacy and confidentiality that integrated services provided:

They started agreeing to be tested, because whenever they go inside, nobody will know the services she is getting inside the room. Before the services were not integrated, they feared. (Registered Nurse, Hospital, Kitui)

As cited earlier, health providers reported that many clients openly expressed gratitude to them for changing the format of service delivery.

In terms of operational convenience, most providers reported that they no longer have to move from room to room serving clients, as they now provide the same services in one room. They also reported that facilities now lose fewer clients particularly due to reduced number of internal referrals. Before integration, clients were often referred from provider to provider and would encounter queues at each door; thus most of clients used to leave before seeing the next provider, discouraged by the queues.

Clients…don’t have to queue 3 or 4 times in the same visit now. They are happy now. (Registered Nurse, Hospital, Nyeri)

In addition, under-staffed health facilities tend to suffer less under integration due to the reduced number of ‘service points’, as the few available staff can provide a wider range of services to more clients.

We are still few staff now, (but because) services are integrated like that, we are able to provide more services and to see all the clients. Not like before. (Registered Nurse, Sub-District Hospital, Kitui)

In direct contradiction to the findings on increased workload discussed later, a few providers said that integration had actually significantly reduced their workload. Some of those that reported reduced workload cited reasons that were more about new resources invested to support implementation of integration, and these included: ability to prescribe long-term FP methods (which meant fewer return clients); and increased numbers of staff due to new recruitment. However, some of the reported reduction in workload in certain facilities was induced by integration itself. For instance, the fact that providers in each facility were now providing a similar profile of services implied an efficient re-distribution of client-load per provider, compared to previous vertical services when each provider served, potentially, the total client population.

Before (integration), they used to say MCH is for pregnant mothers, the postnatal mothers (and so on)…But nowadays (clients) can go anywhere. Maybe a client may go (to MCH) for family planning and at the same time the client is sick. It means she will also be treated and everything is finished there; so I might not get a long queue because most of the clients are finished there and they are given the service there and they walk out. (Clinical Officer, Sub-District Hospital, Kitui)

### Challenges to effective integration

Table 3 summarises the challenges to effective integration reported by providers.

In spite of positive staff demeanour about integration and its well acknowledged benefits, providers at some facilities admitted the presence of significant challenges to effective delivery of integrated HIV and reproductive health services. Challenges to integration can also be viewed at individual and operational levels.

#### Poor work conditions and support

There was a sense that although most providers reported gaining satisfaction and motivation from being able to serve clients better under integration, basic conditions of service remained a source of concern for many of them. One of the key sources of dissatisfaction was the salary which many said lagged behind workload delivered. On bringing up the topic in the interview, some of the respondents expressed irritation, not wanting to discuss the subject further.

[*Laughing*] Oh my God! I’m not satisfied! You realise that the salary you are getting, although we say that nursing is a calling, at times you may not even (meet) your needs. (Registered Nurse, Hospital, Nyeri)

If you compare what you are really doing and the returns, you find that they are not equitable. (Registered Nurse, Hospital, Thika)

Many providers expressed concern that poor remuneration frequently distracted individual commitment to duty. Some of them had come to surrender to the poor conditions, and drew solace only in the fact that it was a system-wide problem.

A second major concern was lack of psychosocial support for providers to help them cope with occupational stressors: ‘burn out’ from increased workload per provider, as well as severe clinical cases that they deal with. Severe cases include those related to HIV/AIDS, extreme poverty, and domestic violence.

Sometimes you meet extreme cases that really leave you crushed…not able to cope. Sometimes the situation (you are dealing with is so severe) you ask yourself how that could happen to a human being… (Registered Nurse, Hospital, Thika)

While at some health facilities supervisors were reported to organise occasional unwinding meetings for staff, most facilities did not have any form of psychosocial support. Majority of health workers reported reliance on prayer, improvised exercise, and in some cases sharing with their partners at home after work.

You are going home, but you are still wearing that coat… You feel you are going home with what you experienced during the day. (Enrolled Nurse, Health Centre, Kitui)

#### Lack of systems adaptation to support integration

Even though some providers reported that integration had reduced their workload, the facility and systemic conditions that facilitated that positive impact on workload did not seem to exist in every *Integra* study facility. As mentioned previously, in the majority of study facilities, integration of services had brought with it increased workload. Increase in client numbers attending the facility, as reported by providers, and the task-shifting effect of integration on individual providers, seemed to be the two main causes. It is important to recognize the uncertainty around whether the increase in client-load per provider was due to actual net increase in service uptake; was merely an effect of client re-distribution among providers due to integration of services; or possibly a combination of both.

For task shifting, providers explained that offering more than one service per client meant that each provider was experiencing an expanded client register as they now received a broader range of clients than in the previous vertical service provision format.

The nurse who is there now has a lot to do, she spends a lot of time with the client because she has to provide (more than one service) to the client. Before, (workload) was less… (Enrolled Nurse, Health Centre, Nyeri)

Many providers reported frequently working overtime to meet the increased demand – without any guarantee of compensation for the time. The workload per provider was compounded by the amount of clinical reporting that necessarily accompanied increase in client numbers per capita but remained separated by service resulting in duplicate reporting for a single client who received several services.

It is a challenge because you find that you have so many registers, like now you find that you have separate STI register, you have the FP register, you have the post natal register, so it is a challenge to (make entries) in all those books for each client…(Registered Nurse, Hospital, Nyeri)

It is tedious for the nurse doing it, patients are there waiting and yet she has to write the reports… (Registered Nurse, Sub-District Hospital, Kitui)

Some providers reported well adjusted clinical recording systems that were integrated to enable efficient reporting at facility level. However, majority of providers reported that clinical case recording was problematic. The result is that, although staff fully appreciate and value the need to report, the quality of recording is significantly compromised.

Sometimes people don’t record anything, or they record poorly. (Enrolled Nurse, Health Centre, Kitui)

Furthermore, the increase in client numbers per provider was reported by many to have increased both waiting and session times at each provider in a facility. In integrated health facilities, provision of more than one service per client almost inevitably increased session time per client which, in turn, raised waiting time for clients waiting for their turns outside the consultation room. Despite the fact that the number of queues a patient had to wait in was reduced, providers still felt waiting times were discouraging. Providers reported receiving complaints from clients because of long waiting and session times, and sometimes impatient clients left before their turn although such ‘protest’ behaviour did not seem to be common experience among providers.

They complain that we are keeping them (waiting) and yet when you are with a client, you must give that client the integrated service. But…they feel that you are (unnecessarily) keeping them waiting (outside). We are still educating them… (Registered Nurse, Sub-District Hospital, Thika)

When a mother comes to my postnatal clinic… If I’m having a client inside and they’re in a hurry, they go. So I don’t get to see most of my postnatal clients. So that is a challenge. (Registered Nurse, Sub-District Hospital, Kitui)

Providers reported an apparent lack of clear guidelines for charging service user fees in the new context of integration. Charging clients a user fee is standard policy in public health facilities in Kenya. Providers reported the uncertainty around charging user-fees for non-index services that were offered to clients who had come for something else. In many facilities, collecting a fee for a single service when the client was receiving multiple services in a contact seemed uneconomical; yet, increasing the fee had the potential to depress demand for services at the facility. Thus, along the way, many services were threatened due to the consequent inadvertent cross-subsidization that occurred between different newly integrated services. This seemed to be compounded by the increase in client numbers and service uptake due to integration.

Direct FP outpatients pay 20 Shillings for the (FP service). But clients who come for other services we are supposed to offer them FP as well and we don’t know whether to charge them, so (it means) we have so many other FP clients who get these services for free…which are paid for by few. So…then you wonder, who is supposed to source for this money to purchase the essential commodities to have all these offered services running (smoothly). (Clinical Officer, Health Centre, Kitui)

Finally, almost all the providers also reported generic health systems constraints including persistent lack of appropriate medical equipment, inadequate drugs and other supplies, and inadequate consultation room space. Some providers reported erratic electricity and water supply. This can be difficult for providers sometimes when, for instance, they have to tell an AIDS patient that the drugs they need are not available.

The challenge there is like when she gets here and she is told that these drugs are not there and yet out there they are told that HIV drugs are free, so (sometimes you) feel for them… they will think that we are (lying to) them. (Enrolled Nurse, Hospital, Thika)

## Discussion

The goal of this qualitative study was to gain an in-depth understanding of frontline health workers’ ongoing experience with providing integrated HIV/AIDS and reproductive health services, and identify benefits and challenges faced by providers. In practice, providers reported delivering services in provider-level and unit-level integration, as well as a combination of both. The summary of benefits and challenges in Table 3 reveals a mixed experience of integration by providers in the study facilities at individual and operational levels.

Health care providers reported individual level benefits of integration that suggested many had successfully made the transition to integrated delivery. These included, at psychological level, job satisfaction and professional stimulation; but also, at more substantive level, providers felt they gained through enhancement of their skills and the broadened scope for experiential learning. Skill-enhancement and experiential learning opportunities that integration brings to a provider role through expansion of responsibilities has been reported in other previous case-studies [18,19]. However, these perceived benefits and motivational factors were countered by challenges that need to be dealt with if motivation to transform service delivery is to be maintained. In particular, many providers felt that their salaries were poor and incommensurate with their new set of integrated tasks and the associated workload. Many also suffered from increased occupational stress from treating severely ill, abused and poor clients, with no formal psychosocial support to help them cope. Majority said they improvised to ‘stress-relieve’ themselves. However, some providers reported that their supervisors organised occasional unwinding meetings to discuss occupational issues including stress, and the meetings seemed to work. Formalising regular debriefing or ‘*unwinding*’ meetings may provide an immediate and practical solution to occupational stress among staff in needy facilities.

At operational level, existing literature highlights a consistent list of issues relating to health service integration: workload, waiting times, contact session times, human resources, physical room space, equipment, drugs and other medical supplies [1,8,10,13-15]. While workload, waiting times, session times and human resources primarily pertain to the client-provider interaction; the rest of the issues represent ‘inputs’ or ‘resources’ that support the interaction to produce an effective and efficient integrated service. Both ‘client-provider interaction’ and ‘input’ issues were widely reported in this study too. Yet, as Table 3 indicates, operational level benefits were reported as well, notably: increased repeat visits and service uptake, increased willingness among clients to take an HIV test, and reduced loss of clients. These benefits, particularly, have been underscored as the core virtues of integration. Arguably, the certainty of these benefits may not be easily established without quantitative triangulation. However, reports of integration benefits represent positive provider perceptions about how well a new system is performing and these perceptions should be taken seriously and seized upon. Perceptions drive provider behaviour, which is central to the long term success of change and integration [11,20].

A note about *workload* in relation to integration: health workers reported workload as being both attenuated and aggravated by integration. In literature, the general observation is that service integration tends to increase workload for the provider in the facility. Several factors have been associated with that increase in workload. For instance, in Ghana [14], South Africa [13], Kenya and Ethiopia [21] increase in workload was found to be driven mostly by increased service uptake in the context of inadequate human resources. Another study in Ethiopia found that increase in workload during integration may also be due to expanded client-provider protocol that increases session times per contact with each client [8]. Both factors were reported in our study, with expanded protocol being the most associated with long waiting times due to increased session times per client. Our study directly suggests some solutions to the problem of increased workload and these were highlighted by providers who reported that integration had *reduced* their workload. Some providers attributed reduced workload to investments in human resource numbers ahead of integration; or changes in prescription policies especially in family planning counselling where emphasis has been more on long-acting contraceptives which translates into fewer clients returning in the short-term for ‘re-fills’ . Investment in human resource numbers reduced workload through client-load re-distribution, whereby previously uneven client-loads were now shared more evenly among a greater number of providers who offer more multiple services. With the potential that client-load re-distribution may lead to reduced queue-lengths per provider, managing workload may be key to mitigating the problem of long waiting times reported in this and other previous studies [19,22]. Both workload and long waiting times may also be addressed through managing client appointment times such that a reasonable number of clients are scheduled to visit each provider in the health facility on each day.

Finally, this study confirms previous studies on the impact of (usually pre-existing) service-guideline, infrastructural and logistic deficiencies (local service policies, physical space, information systems, equipment, drugs and other medical supplies) on the delivery of integrated services [1,14,23]. Two specific examples just to illustrate: First, there were reported ambiguities surrounding the user-fee cost-sharing schemes currently in force which were unclear post-integration (something that has not been highlighted in the literature before); many providers called for clarity of user-charge policy for integrated services. Second, clinical recording procedures were fragmented still in pre-integration format, which compounded the workload challenge for providers and likely compromised integrity of the clinical information system, especially as a source of information for decision-making. Many of the providers recommended a single integrated clinical register for all services provided to a client in a session; and previous similar studies have identified efficiencies gained from integrated clinical information systems in an integrated service context [19,22]. But, beyond the effects of these (infrastructural and logistic) deficiencies in themselves, our findings illustrate the potential negative impact on provider motivation.

The promotion and introduction of integrated health services, like any form of organisational change, generates expectations in both providers and their clients. Thus, for providers resource shortages and weak support systems may be a source of occupational frustration and could affect their morale and overall productivity [24]. Because these problems tend to lie beyond the scope of facility-level decision-making, central level policy decisions need to take them into account right at the point of adopting integration as the new service delivery strategy in the health system. This qualitative study has helped highlight the fact that both pre-change and post-change phases are crucial to the success of integration as a service provision format at frontline health facilities. Central level health policy must lead the process of change to ensure that service delivery support systems and structures are adapted to the needs of integration; infrastructure is upgraded to ensure availability of sufficient room space in health facilities; and that medical supplies, utility services, and human resources are consistently sufficient; in addition to managing initial provider anxieties and other barriers to successful change. Once these have been addressed and the transformation accomplished, a subsequent focus should be on how to manage the post-change phase to ensure sustainability of the transformation. Previous work has noted the importance of commensurate staff incentives and benefits ‘packages’ [9,12,25] and that good management of the transformation process is key to sustaining successful change [11,12,26] and effective delivery of health services after transformation [14,21].

What was not done in this study, which might be attempted in future, is the linking of provider experiences at individual level to the model of integration in operation and actual performance of their facility. This study focussed on bringing out the issues; more complex linking analyses will be the next logical step.

## Conclusion

The success of integration is primarily contingent on the performance of service providers which, in turn, depends on a whole range of facilitative organisational factors [25]. The perspective of providers on the experience of integration is, therefore, critical to ensuring that integration delivers on its promises as well as understanding how it may fall short. This qualitative study was based on that premise. Within the broader context of *Integra* project, the aim for indepth interviews with providers was to generate qualitative data to complement quantitative findings from other elements of the research project. However, the findings also speak to integration programmes beyond *Integra* study facilities in which the interviews were conducted.

Provider experiences from this study demonstrate that successful integration requires a health system-wide commitment at both planning and implementation stages [1,9,10,23,27]. The central Ministry of Health needs to create a coherent policy environment, spearhead strategic planning and ensure availability of resources for implementation at the lower levels. Health facility staffing norms, technical support, costing and reporting procedures, salary and incentive schemes, clinical supply chains, and resourcing of health facility physical space upgrades, all need attention. Most of these are generic health systems issues, but they affect delivery of integrated services. The confusion over fee-charging and the need for integrated reporting registers are two systems issues that our study has highlighted that particularly impede effective delivery of integrated care.

Despite these systems challenges, this study has shown that integration of reproductive health and HIV services can have a positive motivating effect on staff and can lead to better sharing of workload – these are important opportunities that deserve to be built on.

## Competing interests

The authors declare that they have no competing interests.

## Authors’ contributions

RM conceived of the study idea, analysed the data and drafted initial manuscript. SM conceived of the study idea, analysed the data and critically reviewed the manuscript. MC analysed the data and critically reviewed the manuscript. JB reviewed and edited the manuscript. JK coordinated data collection fieldwork. CN coordinated data collection fieldwork. All authors read and approved the final manuscript.

## Pre-publication history

The pre-publication history for this paper can be accessed here:

http://www.biomedcentral.com/1472-6963/13/18/prepub
